# A rare case of IgA lambda multiple myeloma in a 32-year-old woman with t(14;16) translocation associated with kidney injury and non-albumin proteinuria

**DOI:** 10.1186/s12882-024-03600-3

**Published:** 2024-05-16

**Authors:** Ranim Razzouk, Nour Khattab, Maysaa Hoteit, Hala Kfoury, Mustafa Saleh, Bassem Tanios, Jean El-Cheikh, Samir Mallat

**Affiliations:** 1 Department of Internal Medicine, Division of Nephrology and Hypertension, American University of Beirut Medical Center, Beirut, Lebanon; 2 Department of Pathology, American University of Beirut Medical Center, Beirut, Lebanon; 3 Division of Hematology/Oncology, Department of Internal Medicine, American University of Beirut Medical Center, Beirut, Lebanon

**Keywords:** IgA lambda, Multiple myeloma, TP53 extra copy, T(14;16) translocation

## Abstract

**Background:**

Multiple myeloma (MM) is a malignant disorder characterized by monoclonal differentiated plasma cells. While it is more commonly diagnosed in elderly individuals, it can also affect younger populations, though with a lower incidence.

**Case presentation:**

Here, we present the case of a 32-year-old woman diagnosed with IgA lambda MM. She presented with fatigue, nausea, acute kidney injury (AKI) with a rapid increase in creatinine, and anemia. A kidney biopsy was done to rule out a rapidly progressive glomerular disease and a diagnosis was thus reached. A genetic workup revealed t(14;16) translocation and an extra copy of TP53. The patient received aggressive intravenous steroids and intravenous fluid resuscitation, resulting in an improvement in renal function. Treatment with daratumumab in combination with bortezomib, thalidomide, and dexamethasone was initiated and well tolerated. Despite the generally poor prognosis of IgA MM, our case emphasizes the importance of considering MM in young patients with unexplained kidney injury.

**Conclusion:**

Early recognition and prompt intervention are essential in managing MM patients, especially in those with high-risk cytogenetic abnormalities. This case serves as a reminder for clinicians to maintain a high index of suspicion for MM, even in younger populations, when presented with unexplained kidney injury.

## Introduction

MM is a malignancy of monoclonal differentiated plasma cells and is a subclassification of monoclonal gammopathies [1]. It is the second most prevalent hematological malignancy worldwide following Non-Hodgkin lymphoma [2–4] and usually attains men with a mean age of 65 years [2]. This, however, does not prevent the younger population from being affected, with an incidence of less than 3% in those who are younger than 40 years of age [3].In MM patients within this age group, the incidence of IgA MM is not very common [5] and varies between 4.5 and 21% according to different studies [5].

Two scores are usually used to determine the prognosis of newly diagnosed MM. One of those tools is the International Staging System (ISS) for MM [6]. It is based on serum albumin and beta2-microglobulin. In accordance with the International Staging System (ISS), 29% of patients were classified as ISS-1, 38% as ISS-2, and 33% as ISS-3 ^6^. ISS-3 patients exhibited a significantly higher prevalence of kidney impairment and was associated with a higher occurrence of stage 4 or 5 chronic kidney disease (CKD), with rates of 23% and 20%, respectively [6]. This contrasted with patients classified as ISS-2 (6% and 1.5%, respectively) or ISS-1 (4% and 1%) [6]. Another tool is the Revised International Staging System (R-ISS) [7]. It utilizes serum albumin level, beta2-microglobulin, lactate dehydrogenase, and genetic markers. It serves as both a prognostic and predictive tool for early relapse (within 24 months) following autologous transplantation in individuals newly diagnosed with MM [7]. Our patient had an ISS as well as an R-ISS of stage 3.

Herein, we report the case of a 32-year-old woman who presented with fatigue nausea, and acute kidney injury (AKI) with a rapid increase in creatinine and anemia. To our knowledge, this is the first case of reported IgA lambda MM in a woman less than 40 years of age.

## Case presentation

### Patient information

A 32-year-old woman presented to the emergency room for a one-month duration of nausea, fatigue, and recent onset of vomiting. Blood tests done at a peripheral hospital 3 days before presentation revealed AKI (creatinine of 2.3 mg/dL) and anemia. Intravenous fluids were administered without improvement of kidney function so she presented to our hospital for continuity of care.

Upon interview, the patient had a self-resolved upper respiratory tract infection two months earlier. She reports a recent onset of foamy urine with no other urinary symptoms. No recent weight loss, fever, chills, night sweats, arthralgias, bone pain, skin changes, easy bruising, or lower limb edema. She has no personal or family history of kidney disease. She reports rare use of NSAIDs and paracetamol as needed only. Her last creatinine was 0.6 mg/dL back when she was pregnant with her only child one year ago. The course of pregnancy and delivery was uncomplicated. No major interim events occurred since then.

### Clinical findings

The physical exam was unrevealing. Vitals signs were stable. Her blood pressure was 117/79 mmHg, heart rate 84 beats per minute, and temperature 36.7 degrees Celsius. Importantly, there were no rales or lower limb edema. No rashes, bruises, or petechia were noted.

### Diagnostic assessment

Laboratory findings on presentation are shown in Table 1.

Ultrasound of the kidneys showed bilateral increased cortical echogenicity, suggestive of renal medical parenchymal disease with the right kidney measuring 13.8 cm in length and the left 14.6 cm in length. No hydronephrosis or stones were present.

**Fig. 1:**
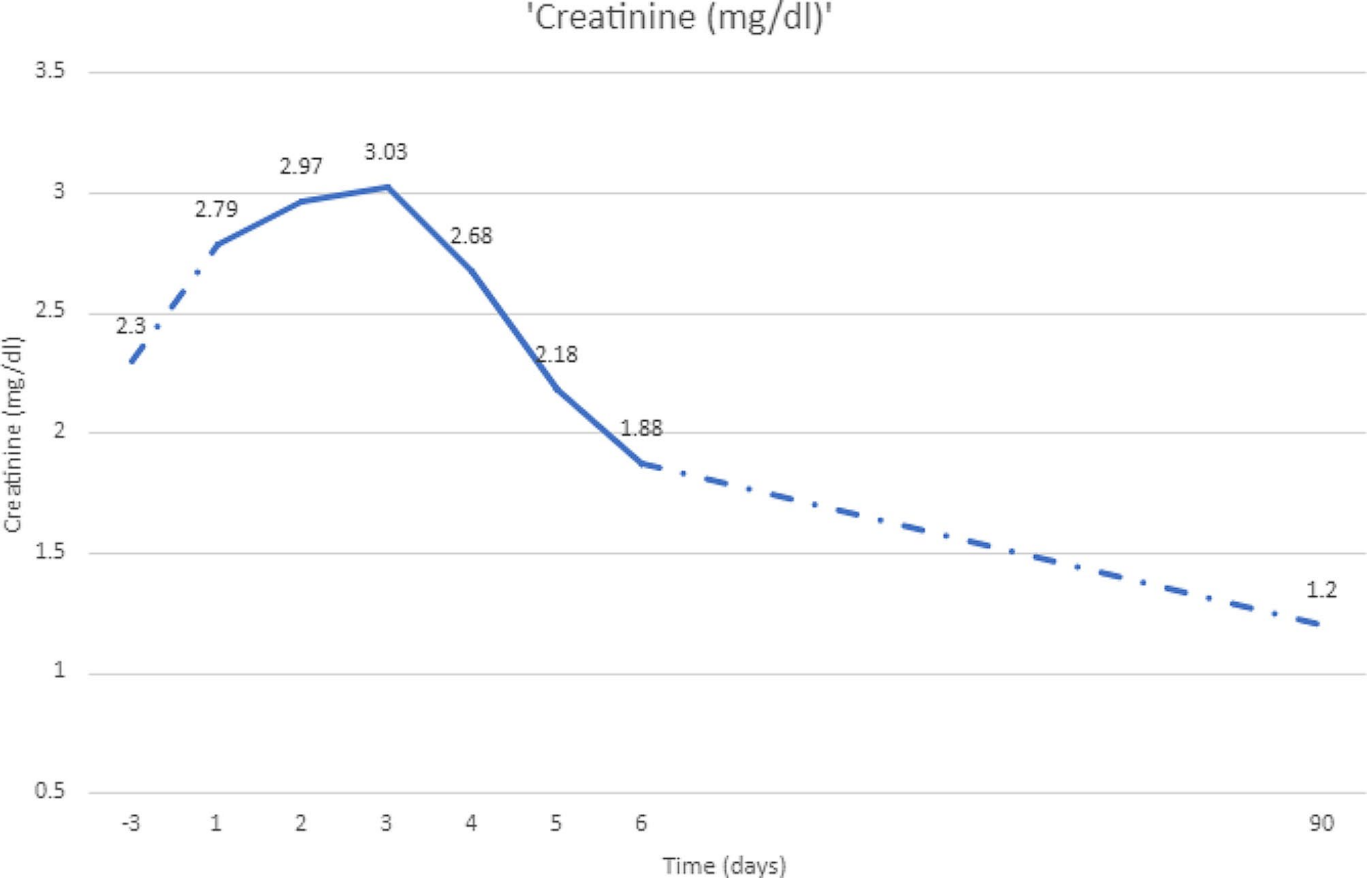
Creatinine trend with a biopsy done on day 3

Considering the rapidly progressive and unexplained kidney failure, and hematuria, (Fig. 1), the decision was to perform a kidney biopsy to rule out a rapidly progressive glomerular disease.

**Fig. 2:**
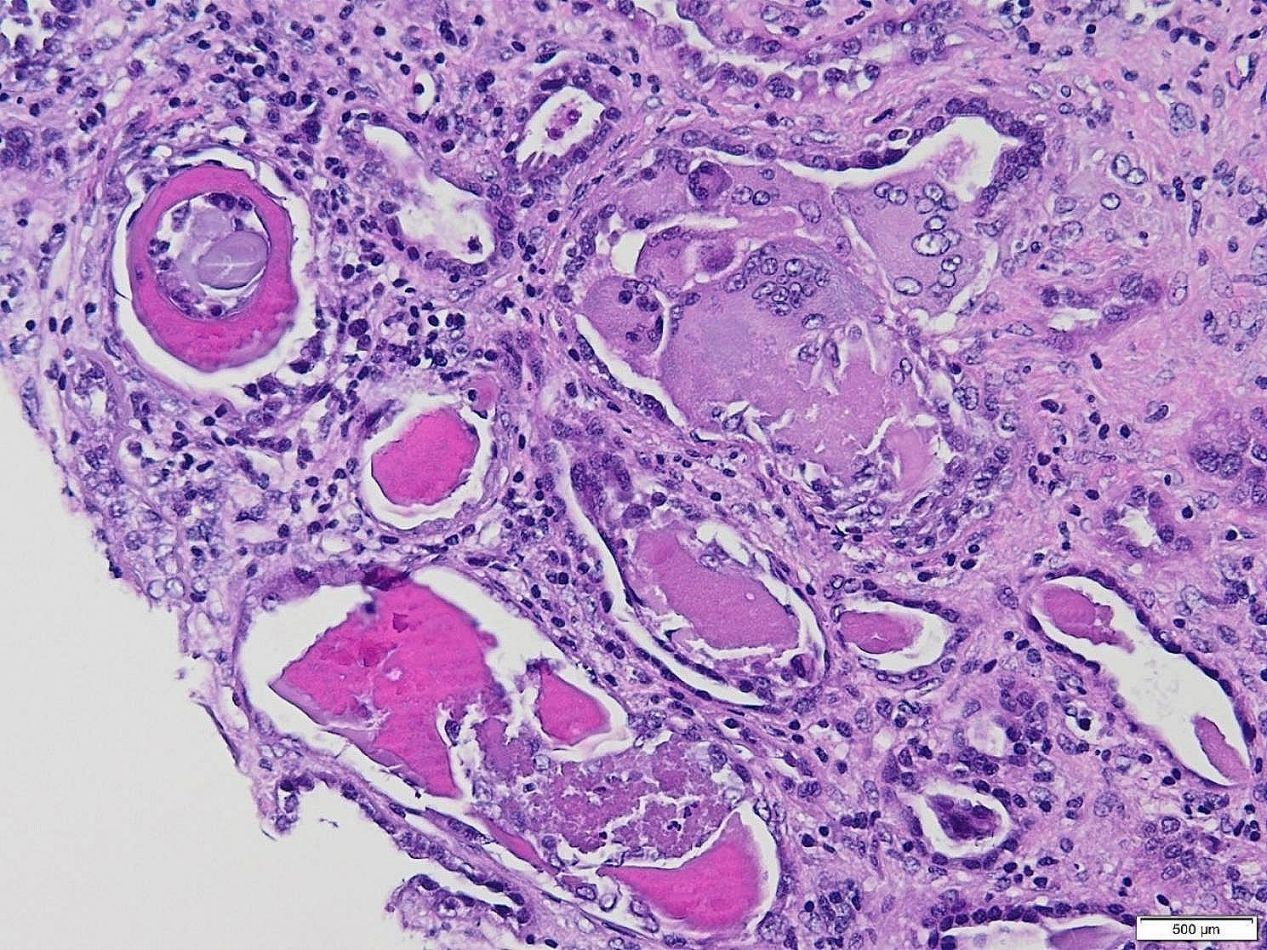
Photomicrograph showing distended tubules with intratubular eosinophilic rigid casts enclosed by multinucleated giant cells, (H&E, x 200)

**Fig. 3:**
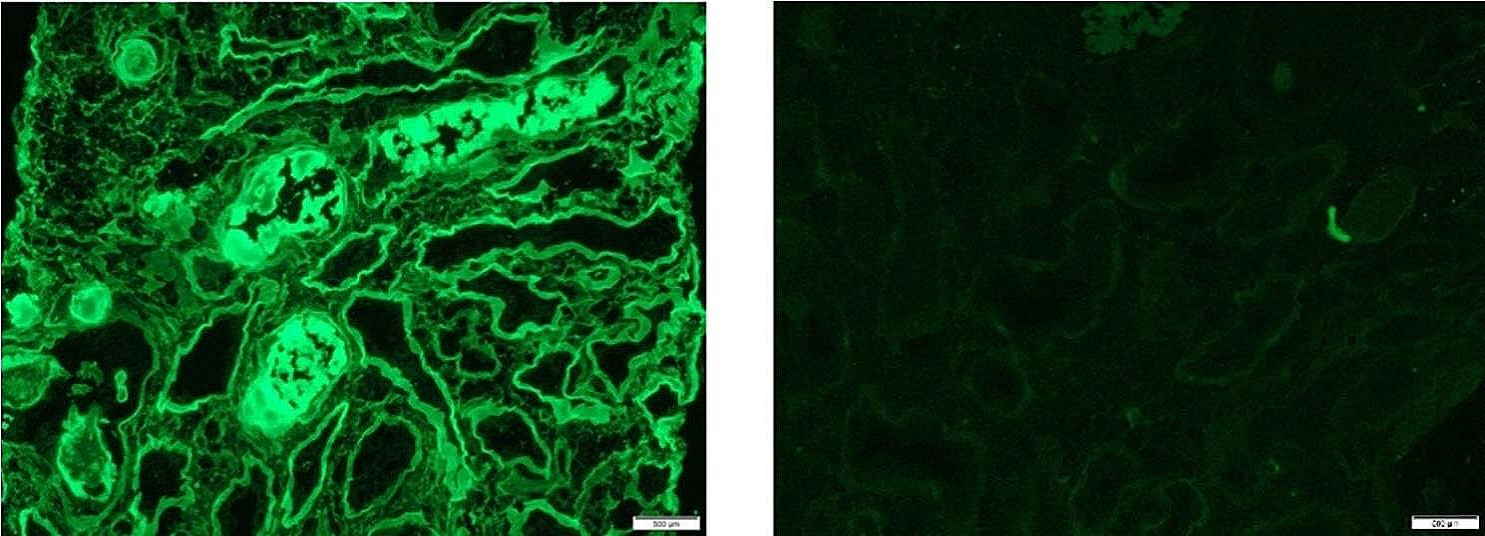
Immunofluorescence staining highlighting the strongly positive lambda light chain in the tubules while kappa is negative (x200)

The kidney biopsy is shown in Figs. 2 and 3. It showed myeloma cast nephropathy. Up to six (6) glomeruli are identified in representative sections showing mild mesangial hypercellularity. Many tubules were plugged with eosinophilic PAS-negative fractured angulated casts with histiocytic reaction and lined by reactive/regenerative tubular epithelial cells. There was an associated acute tubular injury and mild tubular atrophy and interstitial fibrosis, involving approximately 30% of the sampled cortex. Patchy infiltration by chronic inflammatory cells was noted in the interstitium. There is no arterial intimal fibrosis nor arteriolar hyalinosis. No vasculitis or fibrinoid necrosis was noted. The Congo red stain was negative.

A few days later the paraproteinemia workup came back positive for IgA lambda monoclonality in both serum and urine. The free light chain ratio was 0 with a lambda level of 5064 mg /L and a kappa level of 10 mg/L. Beta 2 microglobulin was 15 mg/L.

**Fig. 4:**
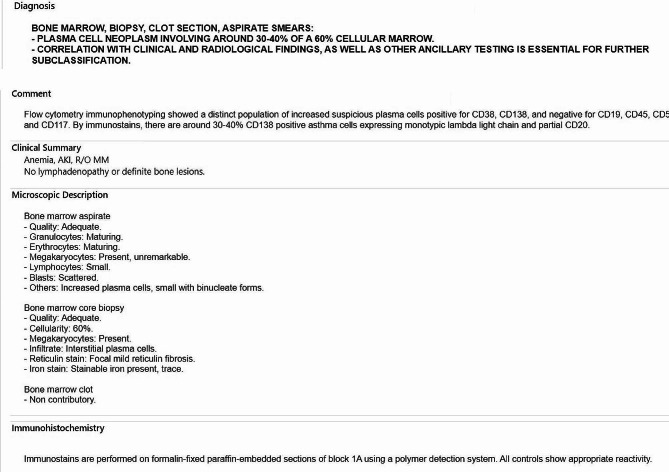
A and B Bone marrow

**Fig. 5:**
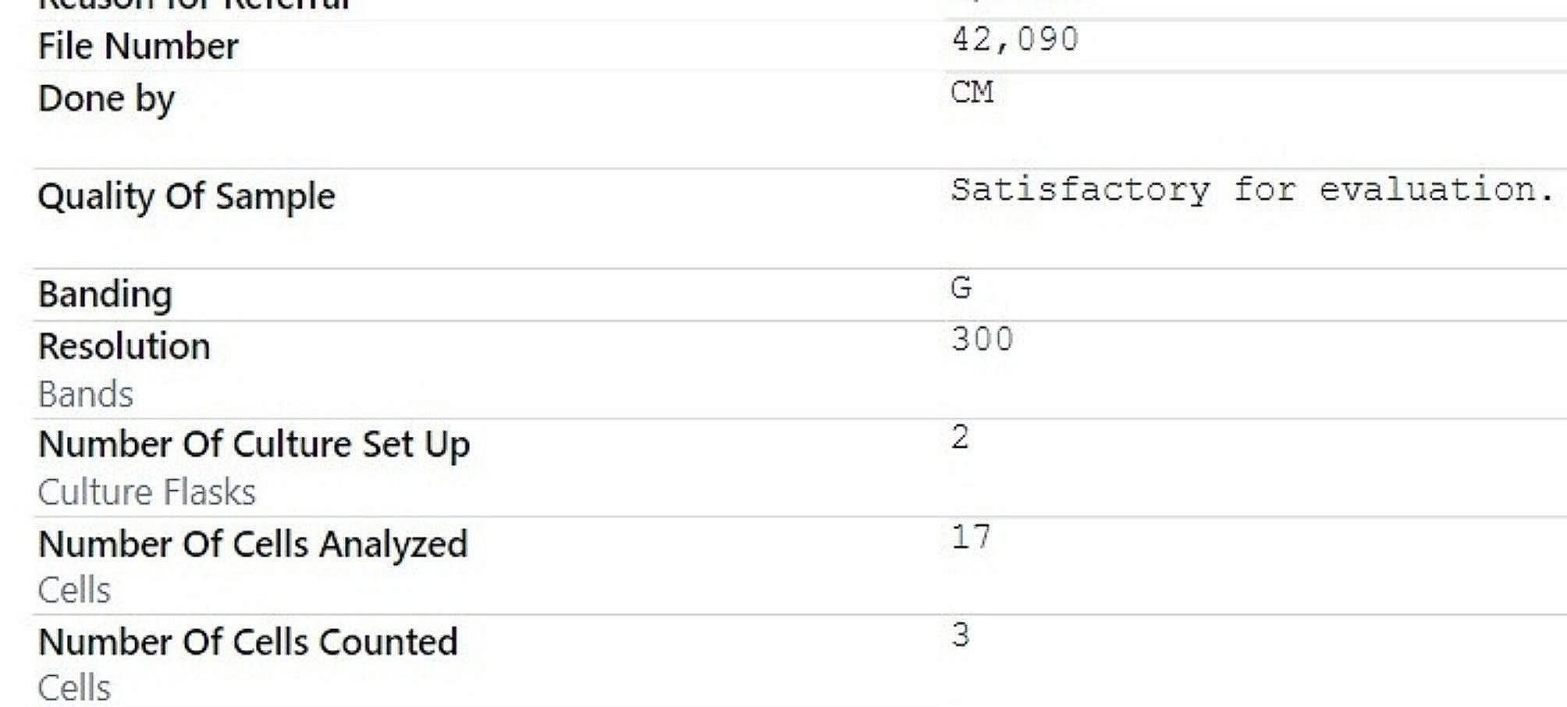
Karyotype

**Fig. 6:**
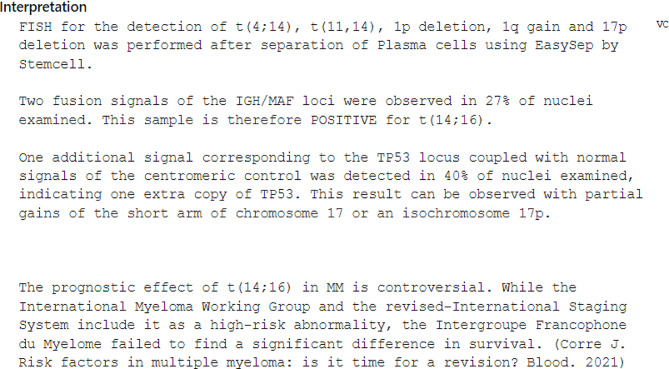
Fluorescence In Situ Hybridization (FISH)

Bone marrow biopsy was done and was pertinent for a low level of metamyelocyte of 4% and an elevated plasma cell level of 23%. The bone marrow and karyotype are shown in Figs. 4 and 5. A total body magnetic resonance imaging revealed no focal bone lesions. A genetic workup revealed a positive sample for t (14;16) and one extra copy of TP53 ( Fig. 6). No evidence of trisomies.

The patient was thus diagnosed with MM with Lambda IgA light chain predominance.

### Therapeutic intervention

After the kidney biopsy, the patient was started on high-dose intravenous steroids with aggressive intravenous fluid resuscitation. Her creatinine started to decrease thereafter.

She was then referred to hematology and treatment with daratumumab in combination with bortezomib, thalidomide, and dexamethasone was started.

### Follow-up and outcomes

The patient tolerated her treatment well. However, after the second cycle, she contracted a COVID-19 infection and received Remdesivir for 5 days with an unremarkable course of illness. Three months later, her creatinine value is 1.2 mg/dL and free lambda light chain levels have normalized to a value of 15.43 mg/dL. She was referred for bone marrow transplantation after 4 cycles of chemotherapy. At the last follow-up, she is 4 months post tandem autologous stem cell transplant (ASCT) in complete remission on maintenance therapy.

## Discussion

The youngest reported case of MM is an 8-year-old boy who presented with anemia, AKI, and lytic bone lesions and was later diagnosed with IgG MM in 2014 [8]. The most common type of myeloma protein (M protein) is IgG at a rate of 52%, with Ig A ranking as second at 21%, Ig D at 2%, and Ig M at less than 1%. Light chain-only disease occurred at a frequency of 16% only [9] with kappa light chains being produced four times more than lambda light chains by neoplastic cells [10].

According to a study done by Rao Pydi et al. on 258 patients younger than 40 years of age diagnosed with MM, the most common presenting symptoms were bone pain (59%) and fatigue (45.4%) [5]. A Chinese study conducted by Wang et al. on 129 patients with IgA MM showed that the most common features of Ig A MM were bone pain (63.2%), extramedullary plasmacytomas (31.7%), and pleural effusions [11], none of which was evident in our patient. Further, a French cohort retrospective study reviewed MM patients less than 40 years old throughout 15 years [12]. They found that younger patients exhibit the same disease characteristics as older patients, except that they had a higher percentage of low-risk ISS when compared to the older population [12].

Whether directly caused by the disease itself or from other unrelated etiologies, kidney failure occurs in 25 to 75% of patients [3] and is the most common initial presentation [4, 13]. 1 to 10% of patients require dialysis on presentation [13–15] with cast nephropathy being the lesion with the highest dialysis requirements [15]. However, with the novel therapies, dialysis independence can be reached in up to 50% of patients [14, 15].

The most prevalent cause of renal failure is immunoglobulin nephrotoxicity, which may lead to amyloidosis (15–35%), light chain deposition disease ( 20–25%), and most commonly cast nephropathy (40–63%) [3]. The latter confers a more acute kidney injury compared to a subacute or progressive course in other etiologies [16].

Light chain cast nephropathy (LCCN) or myeloma kidney, typically presents in more advanced stages of MM and is not associated with any particular light chain [3]. The formation of casts is typically mediated in the distal tubule as a result of light chain and uromodulin complex formation [4]. It may be potentiated by multiple factors including volume depletion such as hypercalcemia or diuretic use, infections, nonsteroidal anti-inflammatory drug (NSAID) use, IV contrast (the prevalence of contrast-associated AKI is 0.6–1.25% in patients with MM), acidic urine, high urine sodium level, and renin-angiotensin blockers [3, 4, 16]. It is also directly proportional to the concentration of free light chains (FLC) ( usually more than 1000 mg/dL) [3, 4] as seen in our patient. Also, LCCN constituted the most common pathology in 40–60% of patients who underwent a kidney biopsy [3]. Biopsy findings are typical for casts in the distal tubules and collecting ducts, with irregular and lamellated shapes that are often cracked [4]. In about two-thirds of cases, a hallmark finding is cast surrounded by giant cell reactions [4]. This was exhibited in our case as well. Only LCCN is considered a myeloma-defining event compared to the other lesions [17]. The proteinuria is usually a Bence Jones proteinuria with < 10% albuminuria [17].

It’s advised to conduct a renal biopsy in individuals exhibiting substantial albuminuria to eliminate the possibility of AL amyloidosis or monoclonal immunoglobulin deposition disease (MIDD) [18]. In fact, Ecotière et al. noted a considerable portion of patients displaying secondary renal conditions [18]. In as many as 70% of cases, the diagnosis of MIDD is made through renal biopsy before identifying dysproteinemia [19]. Additionally, patients with sFLC levels below 500 mg/L should also undergo kidney biopsy as myeloma cast nephropathy becomes improbable [18]. In another study by Nie et al. done in China, patients with monoclonal gammopathy and concomitant AKI were reviewed. They identified 1164 patients with monoclonal gammopathy, 12.9% of whom underwent a kidney biopsy. Among these patients, more than 40% had lesions that were not related to monoclonal gammopathy, with membranous nephropathy being the most common [20].

Due to the significance of renal abnormalities, renal biopsy holds a crucial role in diagnosing and examining AKI in MM patients [18]. However, if a patient has proteinuria with more than 1 g/dL, albuminuria < 10%, and a FLC level of > 150 mg/dL, a kidney biopsy may not be performed as there is a high probability of a LCCN diagnosis [17]. Although we acknowledge that a kidney biopsy is not always required for patients presenting with AKI and high suspicion of LCCN, our patient promptly underwent a kidney biopsy because the primary suspicion was an RPGN, to rule out monoclonal gammopathy of renal significance or non-monoclonal gammopathy related renal lesions. Notably, she ended up having a cast nephropathy.

Kidney injury by itself constitutes a poor prognostic factor [16] and renal outcome significantly impacts survival [13]. Older studies showed that patients with Kidney injury have an overall survival between 8.6 and 18 months versus 26 to 34.5 months for patients with normal kidney function [16]. Thus, urgent intervention is needed when kidney injury occurs to ensure renal recovery. This implies vigorous volume repletion with hypotonic solutions and high flow urine output ( 3 or more liters daily), high dose corticosteroids, chemotherapy, and if needed, extracorporeal removal of FLCs [4, 16]. It was shown that renal response correlated with FLC reduction at day 21 [13], as a 60% reduction in FLC at day 21 correlated with a renal recovery rate of 80% [13]. Other predictors of kidney response include tubular atrophy, the number of casts, and the baseline glomerular filtration rate (GFR) [13]. As such, baseline eGFR > 30 mL/min/1.73 m2 correlated with the probability of renal response [13].

Another key component in the treatment of MM is glucocorticoids [4]. Their effects include decreasing FLC formation and deposition, and antagonizing renal edema caused by tubulointerstitial fluid accumulation [4]. However, the cornerstone of MM treatment remains chemotherapy [21]. This serves to decrease tumor burden and consequently reduces nephrotoxic effects [21]. Treatment consists of 3–4 cycles with VRd ( bortezomib, thalidomide, dexamethasone) in standard-risk patients or a quadruple regimen Dara- VRd ( containing daratumumab, a monoclonal antibody targeting CD38 + VRd) in high-risk patients [21]. Our patient received the latter treatment. Stem cell harvest is the next step after which patients may either undergo frontline ASCT or resume induction therapy delaying ASCT until first relapse [21].The treatment approach for newly diagnosed symptomatic MM usually depends on risk stratification and eligibility of ASCT [21]. Regarding our patient, she is classified as having high-risk myeloma. Given her young age and absence of comorbidities, she is eligible for tandem ASCT after her treatment regimen, as it improves outcomes in MM with high-risk cytogenetics [22].

Recently, new treatment methods focusing on FLC removal have been proposed. For example, effective removal of nephrotoxic FLCs by extracorporeal techniques (namely high cutoff hemodialysis HCO-HD) has received much interest [23]. It has been shown that 90% of the total FLC volume can be removed by HCO-HD within 3 weeks versus 25% in plasmapheresis [23, 24]. To date, only two randomized controlled trials, EuLITE and MYRE, were performed to evaluate the differences between HCO-HD and standard high flow-HD. Both trials revealed that the HCO membrane combined with chemotherapy treatment exhibited strong clearance qualities for serum FLCs; however, they both failed to demonstrate significant improvement in renal and hematological outcomes [23]. Therefore, their use remains controversial, awaiting further confirmation with larger trials [23].

Multiple factors affect the prognosis of MM including host characteristics, tumor burden, response to therapy, age as well as cytogenetics [25, 21]. vAccording to an analysis done by Ludwig et al. in 2008 on 10 549 patients, younger age (< 50 years) was associated with a longer survival both after conventional treatment and high-dose therapy with planned autologous stem-cell transplantation. Notably, age was found to be an independent risk factor during conventional therapy only, but not after autologous transplantation [26]. Cytogenetics also play a prognostic role as certain mutations are considered high risk [25]. These include translocations such as t(4;14), t(14;16), and t(14;20) and are associated with a poorer prognosis, whereas others such as (11;14), t(6;14) or trisomies confer standard-risk disease [25]. Interestingly, according to the study conducted by Wang et al., it was found that patients with IgA type MM exhibited cytogenetic abnormalities that were more high risk. This can explain the generally poor prognosis of IgA MM [11]. Our patient had the t (14;16) translocation and was thus at a higher risk, however, according to one study, the prognostic value of this translocation was not confirmed [27]. This particular mutation is closely associated with the Lambda isotype and patients who exhibit it have a higher likelihood of having kidney failure as the predominant myeloma defining event on presentation, also seen in our case [25]. Interestingly, it was observed that MM carrying the t (14; 16) translocation may respond well to steroid therapy due to the higher expression of glucocorticoid receptors in this subgroup as compared to others [28].

Another noteworthy genetic marker is the TP53 gene located on chromosome 17p13, a tumor suppressor that controls apoptosis and cell cycle progression [29]. Mutations are rarely present at diagnosis, however, their frequency increases at later stages proving their role in disease progression [29]. A TP53 deletion is associated with a poor prognosis [30]. Interestingly, our patient exhibited an extra copy of TP53, however molecular sequencing was not done.

In conclusion, the correlation between kidney injury and MM is well accepted. We report this case to highlight the diagnostic and prognostic value of kidney biopsies, as well as the importance of keeping an index of suspicion even in young patients who present with unexplained kidney injury.

**Table:** 

Teaching points
- Less than 3% of patients younger than 40 are diagnosed with MM
**-** T(14;16) translocation is considered high risk with a poor prognosis. It is associated with isotype Lambda and kidney failure as a predominant MM-defining event
- TP53 deletion is associated with a poor outcome whereas its amplification has a good outcome
- Kidney biopsy is useful in selected cases only and can play the role of a diagnostic and prognostic tool
- Cast nephropathy is the most common lesion seen on biopsy
- Removal of FLCs by high cutoff hemodialysis may be used as an adjunctive therapy. However, trials on HCO-HD were of small size with different methodologies and their use remains controversial

**Table 1:** Laboratory findings

Adjusted WBC	4,000–11,000 /cu.mm	7,426
HGB	12.0–16.0 g/dL	9.3 (L)
Platelets	150,000–400,000 /cu.mm	213,000
Glucose	76–110 mg/dL	85
BUN	8–25 mg/dL	19
Creatinine	0.50–1.00 mg/dL	**2.79 (H)**
eGFR	> 60 mL/min/1.73 m*2	**22 (L)**
Sodium	135–145 mmol/L	138
Potassium	3.5–5.1 mmol/L	4.6
Chloride	98–109 mmol/L	104
Carbon Dioxide	24–30 mmol/L	**22 (L)**
Anion Gap	8–15 mmol/L	12
Calcium	8.5–10.5 mg/dL	10.2
Phosphate	2.7–4.8 mg/dL	3.5
Urine analysis		**Microscopic hematuria** with numerous RBC, WBC 2–4/HPF, **protein + 1**, pH 7, and specific gravity of 1.0006, no pathogenic casts were seen
Albumin	36–53 g/L	40
Globulin	18–34 g/L	44 (H)
Lactate Dehydrogenase (LDH)	110–265 IU/L	165
Ferritin	ng/mL	350.0
Iron	37–160 µg/dL	58
Unsaturated Binding Capacity (UIBC)	µg/dL	181
Iron Binding Capacity	270–450 µg/dL	239 (L)
Iron % Saturation	15.0–50.0%	24.3
ANA DETECT	Negative	Negative
ACA IgG	< 10.0 GPLU/ml	< 10.0
ACA IgM	< 7.0 MPLU/ml	< 7.0
ds DNA IgG	< 20.0 IU/ml	< 20.0
pANCA (MPO) Abs	< 5.0 U/ml	< 5.0
Anti-ß2 Glycoprotein 1 IgM	U/ml	< 5.0
cANCA (PR3) Abs	See comment U/mL	< 10.0
Haptoglobin	0.30–2.00 g/L	1.58
C3 Complement	0.90–1.80 g/L	1.19
C4 Complement	0.10–0.40 g/L	**0.64 (H)**
Albumin/Creatinine	mg/g Crea	647.6
Prot/Creat Urine Spot	mg/g Crea	7,535
Protein Urine 24 h	g/24 h g/24 h	6.98 6.98

## Data Availability

All data generated or analyzed during this study are included in this published article.

## Abbreviations

AKI: acute kidney injury
ASCT: autologous stem cell transplant
CKD: chronic kidney disease
Dara- VReligibd: (daratumumab, a monoclonal antibody targeting CD38 + VRD)
DTPACE: (dexamethasone, thalidomide, cisplatin, doxorubicin, cyclophosphamide, and etoposide)
FLC: free light chains
GFR: glomerular filtration rate
HCO-HD: high cutoff hemodialysis
ISS: International staging system
LCC: Light chain cast nephropathy
MM: multiple myeloma
NSAID: non-steroidal anti-inflammatory drug
R-ISS: Revised International Staging System
RPGN: rapidly progressive glomerulonephritis
VCD: cyclophosphamide, bortezomib, and dexamethasone
VRd: bortezomib, thalidomide, dexamethasone
